# Expressing Biomedical Ontologies in Natural Language for Expert Evaluation

**Published:** 2017

**Authors:** Muhammad Amith, Frank J. Manion, Marcelline R. Harris, Yaoyun Zhang, Hua Xu, Cui Tao

**Affiliations:** a School of Biomedical Informatics, University of Texas Health Science Center, Houston, Texas, United States; b University of Michigan, Ann Arbor, Michigan, United States,

**Keywords:** Natural Language Processing, Biomedical Ontologies, Knowledge Management

## Abstract

We report on a study of our custom Hootation software for the purposes of assessing its ability to produce clear and accurate natural language phrases from axioms embedded in three biomedical ontologies. Using multiple domain experts and three discrete rating scales, we evaluated the tool on clarity of the natural language produced, fidelity of the natural language produced from the ontology to the axiom, and the fidelity of the domain knowledge represented by the axioms. Results show that Hootation provided relatively clear natural language equivalents for a select set of OWL axioms, although the clarity of statements hinges on the accuracy and representation of axioms in the ontology.

## Introduction

Ontologies are artifacts of encoded knowledge that represent pieces of information in a *subject* > *predicate* > *object* format (e.g., *HPV virus* > *increases risk for* > *cervical cancer*) known as triples. Ontologies aim to represent a defined domain space using interlinked triples, harnessed by machines for further processing or machine intelligence tasks. For ontologies to be machine-readable, special syntax is utilized to encode the interlinked triples. For example, the Web Ontology Language (OWL)^1^, or Resource Description Language (RDF)^2^/ Terse RDF Triple Language (Turtle)^3^ are commonly used to encode the interlinked triples.

As a data-source, ontologies are not immune to errors or inconsistencies. Reasons for the errors and inconsistencies are beyond the discussion of this paper, but the importance of this area of ontology work has been highlighted recently in [1] and [2]. Additionally, most biomedical ontologies on the National Center for Biomedical Ontologies (NCBO) Bioportal do not report any evidence of any evaluation [3].

### Background

Evaluation frameworks are invaluable to knowledge engineers constructing or refining an ontology, and when assessing if a given ontology is fit for use. According to Gómez-Pérez, ontology evaluation falls under two categories: validation and verification [4]. Validation examines the purposeful, extrinsic aspect of the ontology while verification examines the internal aspects (e.g., the quality of terms, graphical structure, etc.).

Evaluation of ontologies typically involves assessment along three axes: Syntax, Semantics, Pragmatics. Both qualitative [5–7] and quantitative [8] assessment of the three axes are possible [9]; a common approach is to enlist subject matter experts to review the ontology artifact. Our focus is on the veracity of ontologies, which we ascribe as a verification-based evaluation. We presume that Subject Matter Experts (SMEs) are integral to the quality of the ontology during development phases. However, for an SME to review and assess an ontology, specifically those with little to no knowledge engineering background, we need to present it in a format that is accessible.

Among the challenges when engaging subject matter experts is the substantial learning curve to help these experts interpret the knowledge representation in the ontology [10; 11], and their lack of familiarity with ontology construction and visualization tools such as those in the commonly used ontology editor Protégé. This is unfortunate as many experts could provide significant input in improving the quality of the ontology. One possible solution is to translate the knowledge in the ontology to human-readable natural language statements. Below, we discuss details of our approach.

We propose that expressing an ontology in natural language is likely to provide a more readily understandable approach to interpret the interlinked triples, and thereby provide a valuable resource when engaging domain experts in working with the ontology. The natural language sentences produced by the Hootation tool (described below) can be used to assess ontologies along all three axes. Syntax can be assessed by determining if statements constructed from the model and expressed in natural language are correct when compared to uses cases and other artifacts of importance within the domain. Semantics can subsequently be assessed by determining if the definitions of labels expressed in the sentences convey an accurate and complete meaning in the context of their intended use, and if the classes, association, attributes and relationships in the sentences are understandable and relevant. Finally, close examination of the sentences produced can reveal pragmatic issues with the ontology such as formal completeness i.e., what may be missing from the ontology, and what cognitive effort on domain experts is needed in understanding the ontology. The latter is sometimes used as a proxy measure for consistency of the ontology.

### “Hootation” Java Library

#### Related Studies

Natural Language Generation (NLG) is an expansive topic that has been the focus of considerable previous research. NLG is one of the two main topics of natural language processing – the other centers on natural language understanding (NLU), which is the focus of much biomedical informatics research. While NLU centers on interpreting free text into data for machines to understand, NLG focuses on interpreting data from the machines into free text or documents for humans to understand. In the context of this study, the emphasis is on transforming triples from ontological models to natural language statements that would help evaluate the knowledge contained in biomedical ontologies. The merits of NLG applications for biomedical ontologies include question-answering, document creation and summarization from datasets, concealing the complexity of the syntax, and ontology evaluation.

An early work in this area was ModelExplainer [12] that generated lines of text from object oriented models. Other relevant work for authoring and NLG applications involved the use of bi-directional Controlled Natural Language (CNL) for OWL 1.1 such as Attempto Controlled English (ACE) [13], Sydney OWL Syntax (SOS)[14], Rabbit [15] etc. However, CNL are compounded by the issues of NLU (ambiguity of text) and NLG (difficult to comprehend for users and limitations the label’s nomenclature). None of what has been described provides a dedicated OWL2-to-NL engine that is portable for application use. However, NaturalOWL employs a basic template approach, but it depends on a separate authoring tool for domain dependent generation [16; 17].

#### Hootation

Our Hootation software library is derived from the natural language generation work by Agile Knowledge Engineering and Semantic Web (AKSW) Research Group [18; 19] developed initially for a semantic web application. The NLG layer harnesses the OWL-API; as initially developed, it supported the translation of 12 logical axioms for OWL2. We added support for 6 additional logical axioms, with plans to add more translation for the remaining axioms. Hootation also utilizes SimpleNLG [20], a state of the art NLG engine that provides flexible APIs to manipulate morphological and syntactical aspects of a generated statement. SimpleNLG also allows the use of the NIH Specialist Lexicon [21] for expanded coverage for medical lexicon, which we have yet to exploit, but an added benefit for biomedical ontologies.

While many NLG applications focus on producing documents or other large bodies of text, an immediate goal is proper translation of each individual axiom to NL statements, so that biomedical experts can rate the veracity of the information and then report on the content quality of the ontology. We intend to integrate the Hootation API library into our continued work to provide a web-based tool for comprehensive ontology evaluation (see Future Direction section for details). Source code and a Java binary library will be available for open source distribution^4^.

## Methods

Our primary objective was to determine whether Hootation could accurately produce natural language from biomedical ontologies in a way that is understandable for subject matter experts. We also wanted to evaluate factors that contribute to or hinder the clarity of the natural language. Java code was developed to interface with the Hootation API library, and output for each of the ontologies was exported in CSV format including, for each natural language statement, the corresponding axiom in OWL Manchester format and the type of logical axiom.

### Sample

Because most ontologies do not use every axiom type available by OWL, three ontologies were used to capture the NL translation for diverse axioms.

The “People” Ontology represents knowledge on the types of people based mostly on familial information. The People ontology is a teaching tool for University of Texas Health Science Center students, used as an introduction to the development OWL-based ontologies and as an introduction to the descriptive logic power of OWL. This ontology is based on descriptive definitions from California Polytechnic State University, in San Luis Obispo, California [22]. The ontology used for this study contained 13 classes, 8 properties, 9 instances (90 total) and a variety of axiom types. This ontology was included because of the simple and universal nature of the encoded information as well as its utilization of various axiom types.

The “Informed Consent Ontology” (ICO) [23] is a preliminary ontology based on the analysis of informed consent templates and blank informed consent forms obtained from two separate Institutional Review Boards (IRB) at the University of Michigan. In its current iteration, ICO focuses on informed consent documents and processes. Consequently, the concepts represented by classes and relations in the work are in the context of informed consent documents, and recommendations for addressing concepts of risk, privacy, and other notions of precepts laid out in US Common Law and medical ethics. ICO is based on the Basic Formal Ontology (BFO) [24], represented in the Web Ontology Language (OWL2), was built on Open Biomedical Ontologies (OBO) Foundry principles [25], and inherits the classes, relations, and axioms from the Ontology of Biomedical Investigations (OBI)[26]. ICO contains 375 classes, including 163 ICO-specific classes and 86 properties. The ontology contains 677 axioms, however many of these axioms are inherited directly from the OBI framework, leaving 183 ICO-specific axioms that are studied in this paper. The Time Event Ontology (TEO) is a derivative of the Clinical Narrative Temporal Relation Ontology (CNTRO) by the Ontology Research Group at the School of Biomedical Informatics (University of Texas Health Science Center) [27]. TEO contains entities and definitions relating to temporal information and their semantic relationships between them. Its intention is to “provide a formal conceptualization of temporal structures in both structured data and textual narratives” and “core semantic components for representing temporal events and relations to enable reasoning capacities in temporal relations.” TEO (version 1.7) contains 156 classes, 51 properties, 8 instances, and 1026 axioms. Similar to ICO, TEO is based on BFO.

### Evaluation Procedures

For each ontology, two persons familiar with the logic of the ontology evaluated the NL expression along three dimensions. First, *Clarity* was scored from 1–3, where 1 indicates the natural language expression of the axiom is clear, unlikely to cause confusion or ambiguity, 2 indicates the natural language expression of the axiom is clear, but there may be ambiguity attributable to the axiom, and 3 indicates the natural language expression is not interpretable. *NL Fidelity to Axiom*, addressed whether the natural language expression demonstrated fidelity to the underlying axiom (i.e. logic was accurately expressed in the natural expression). If this dimension was scored as “yes”, the natural language expression is an accurate reflection of axiom; if “no”, the tool appears to have misinterpreted the logic. *Axiom Fidelity to Domain*, addressed the fidelity of the axiom itself to domain knowledge. Although an evaluation of the fidelity of the axiom to domain knowledge is an assessment of the ontology and not an evaluation of the expression generated by the Hootation tool, it addresses this dimension because both syntactic and semantic issues within the ontology itself sometimes confounded assessments of the clarity of the NL expression. A score of 1 indicated that the reviewer agrees with axiom, 2 that the reviewer disagreess or is uncertain about concepts *or* relationships in axiom, and 3 that the reviewer disagrees or is uncertain about concepts *and* relationships indicated by the axiom. Overall, familiarity with the ontology is important as it yields direct expertise of the intention and construction of the axioms, and adeptness in assessing the translation based on the three dimensions.

Evaluators used online, shareable spreadsheets to record their assessments of each dimension. The first three columns of the spreadsheet presented the axiom type (e.g., SubClassOf, EquivalentClasses), the axiom logic expressed in description logic notation, and the natural language expression generated by the Hootation tool (See Table 1). After reviewing the content of each row, each of the evaluators assigned to the specific ontology recorded their assessment of each of the three dimensions in separate columns. Reviewers were not blinded to each other’s assessments because the goal was informed critique and convergence on evaluations of each natural language expression. Disagreements were recorded if scoring decisions could not be reconciled between reviewers.

In order to explore relationship when the NL statement may be clear but the axiom is incorrect, we utilized IBM SPSS (v23) to calculate Spearman’s rho correlation between the *Clarity* values and *Axiom Fidelity to Domain* knowledge values.

## Results

Interrater agreement was calculated among the raters, and the overall agreement for the aforementioned metrics were 86% for Clarity, 91% for Fidelity to Natural Language, and 90% for Fidelity to Domain. For the People ontology, the agreement was 83%) for Clarity and Fidelity of Natural Language to Axioms, and 98%) for Axiom Fidelity to Domain. Likewise, for Time Event and Informed Consent the agreements were 82%,96%, 88%; and 92%, 93%, 85%, respectively. Overall, there was high agreement with the results of the assessment. We caution that each ontology was independently assessed and consequently, the results do not yield normalized quality data of the underlying ontologies.

The data in Table 2 and Table 3 were aggregated and segmented to comprehensively evaluate the evaluators assessment of Hootation’s results.

Table 2 provides a summary of the overall averages for each of the assessment described earlier. Across the three ontologies, the People ontology demonstrated the best rating for Clarity and Fidelity to Domain knowledge; the Clarity metric rating was 1.19, and Axiom Fidelity to Domain was 1.01. The ICO ontology demonstrated the best NL Fidelity for the axioms (95%)). For the Time Event ontology, the average assessment scores were 1.32 for Clarity, 92% for NL Fidelity to Axiom, and 1.13 Axiom Fidelity to Domain. The Informed Consent ontology average assessment scores were 1.28 for Clarity, 95% for NL Fidelity to Axiom, and 1.36 for Axiom Fidelity to Domain). Altogether, the three ontologies yielded an average Clarity of 1.26, 92% (yes) for NL Fidelity to Axiom, and 1.17 for Axiom Fidelity to Domain. The correlation between clarity and fidelity to domain knowledge reveals a positive, strong linear relationship that was statistically significant (r= 0.76, p<0.0l).

Table 3 presents data segmented by 14 OWL axioms supported by Hootation. Because of space constraints, below we show only the two examples demonstrating the range of metrics observed related to axioms. See https://goo.gl/Br3qQU for full table. The majority of the axioms types were SubClassOf, and others appearing in two or more ontologies were Class Assertion, EquivalentClasses, ObjectPropertyDomain, ObjectPropertyRanges, DataPropertyDomain, DataPropertyRange, DisjointClasses, and SymmetricalObjectProperty. Five axiom types, FunctionalDataProperty, FunctionalObjectProperty, DataPropertyAssertion, Differentlndividuals, and ObjectPropertyAssertion, only appeared in the People Ontology. Not all ontologies used every axiom types, and variations in Clarity, NL Fidelity to Axiom, and Axiom Fidelity to Domain are noted across axiom types. For example, across all three ontologies, more complex axiom types such as ObjectPropertyRange scored much worse on clarity than less complex axioms such as SubClassOf.

## Discussion

The results of this study are encouraging with respect to NLG generation for use in ontology evaluation. Overall, Hootation appears to generate natural language statements with clarity, and fidelity to the axiom.

Problems in clarity included the introduction of mid-level noun phrases that would not be typical in a purely natural discourse between domain experts. For example, the ICO axiom: ICO_0000171 ⊆ ∃ IAO_0000136.ICO_0000064 produced the text *“every answer option text entity is something that is about a study requiring informed consent”*. While technically correct, a more natural English discourse might have been written as *“every answer option text entity is about a study requiring informed consent”*. In general, the introduction of the phrase *“is something that”* in descriptive logic axioms containing an existential restriction of the form “∃ R.C” caused considerable discussion amoung the reviewers as to the clarity of the produced phrases.

Another factor we noted during our evaluation that clearly impacts the usefulness of the produced natural language are the class and object property labels of the source ontology. The choice of appropriate labels agreed on by domain experts during the construction of an ontology is generally considered good practice. Iterative use of Hootation during ontology construction and refinement can assist with this.

While not the primary focus of this paper, the fidelity of the axiom to underlying domain knowledge was noted by reviewers of ICO as an issue. Reviewers noted approximately 41 axioms (the number varied slightly between the two reviewers) were not accurate representations of the underlying domain knowledge. ICO developers intend to target these axioms for review and correction. This is strong evidence that tools such as Hootation are useful and effective at improving the quality of ontologies.

Also, the majority of the axiom NL translations were of the SubClassOf type, which intuitively, should be “easy” to translate. However, due to nomenclature of the labels, the translation was not straightforward, and the results point to some lack of clarity and fidelity to domain knowledge.

The finding of a positive correlation between clarity and fidelity of the axiom to domain knowledge merits further investigation, and suggests that when the axiom fidelity to domain knowledge is less accurate, the clarity of the NL statement also diminishes. For example, the TEO axiom, TEO_0000048 ⊆ TEO_ 0000084, which produced *“every Saturday is a week day*” is technically correct within the context of TEO axioms (Saturday is modeled as subclass of weekday entity), but as a generated statement it could be misleading to human evaluators because Saturday is typically discussed as a weekend day, not a weekday. Future work on this relationship needs to account for the complexity of the axiom as well as the fidelity of the axiom to domain knowledge. For example, an axiom of the general form A ⊆ ∃B (∃C.D) is more complex than an axiom of the form A ⊆ B. We also intend to add an option to the program to address discourse type as typified by the *“is something that”* issue discussed above, allowing production of text more suited to a domain expert.

### Software

Other factors that influenced evaluator ratings were software bugs and the stemming algorithm. The tool does not yet support import of external ontologies, so some NLG statements included unresolved names of entities from external ontologies that were not merged into the ontology file, e.g., “*every duration measurement is an iao 0000032*”, where the iao 0000032 is associated to an entity from the external Information Artifact Ontology. Future work may require that the API automatically downloads and resolves references to external ontologies at runtime. Another problem we noticed was introduced by our utilization of the Porter-Stemmer algorithm, which sometimes unnecessarily decomposed a word to a form that is unrecognizable (Ex. “*data*” became “*da*”). WordNet API software packages offer lemmatization that might be an alternative.

#### Limitations and Future Direction

The Hootation software library was limited to 14 OWL axioms that can be translated to natural language statements, most of which were carried over from the work of previous developers. In the future, we plan on supporting the translation of the full set of axiom types to provide comprehensive translation of OWL axioms to natural language statements.

Also, we did not separately examine the impact on clarity of BFO-based axioms for those ontologies using BFO as upper level ontology. BFO uses a specific realism-based model to provide a framework for building other ontologies against. Evaluation of ontologies based on the BFO framework demands some familiarity of the underlying BFO model and the precise terms used by BFO for representing terms, classes, and relationships. Finally, we recognize the need to consider complexity of axiom types in future studies.

One of the ongoing projects we are engaged in is to develop a web-based tool (“OntoKeeper”^5^)[3] to evaluate published ontologies according to various metrics influenced by [8]. One of the metrics include evaluating the veracity of the ontology from subject matter expert review through an online user interface. To do this, we need to translate the logical axioms to natural language statements to be more “readable” for the experts with little knowledge on how to navigate through an ontology or no knowledge of complexities of the OWL/RDF syntax. The finalization of this work will be to integrate the NL translation component to the web-based tool and publish the API library for future research on biomedical ontology evaluation research.

An impending study will perform an extensive evaluation of specific biomedical ontologies with accomplished biomedical ontology experts to review the NL axioms. That study will also include details on implementation of Hootation and near complete support for majority of the axiom types.

## Conclusion

To address the specific need of generating human-friendly interpretation of ontology axioms in natural language, we introduce Hootation. This software library utilizes a combination of the OWL-API and SimpleNLG, as architected by AKSW, to produce basic natural language statements for 14 axiom types. By translating the axioms into natural language statements, we can enlist the participation of domain subject matter experts who can therefore easily review an ontology without the barrier of learning the complexity of knowledge engineering. In the future, we plan on incorporating this library into our prototype software tool OntoKeeper to add a subject matter expert review component. Overall, this work has potential implications for bridging the gap between the expertise of domain experts and encoded knowledge in machine encoded syntax.

## Figures and Tables

**Table 1 -:** Sample output

Axiom Type	Logical Axiom	NL Equivalent
SubClassOf	ICO 0000062 ⊑ ICO_0000073	every human subject unable to give informed consent is a human subject

**Table 2 -:** Average assessment of ontologies

Ontology	Clarity (*μ,σ*)	NL Fidelity to Axiom (% Yes)	Axiom Fidelity to Domain (*μ,σ*)
People	1.19 (0.42)	90%	1.01 (0.14)
Time Event	1.32 (0.63)	92%	1.13 (0.38)
Informed Consent	1.28 (0.58)	95%	1.36 (0.64)
Average	**1.26**	**92%**	**1.17**

**Table 3 -:** Assessments by Axiom Type (example)

	Axiom Type	
	SubClassOF	ObjectPropertyDomain
**NL Expressions (n)**	306	10
**People**	11	4
**Time Event**	118	5
**Informed Consent**	117	1
**Clarity** (**μ****,σ**)	1.14, 0.52	1.3, 0.73
**People**	1.00, 0	1.00, 0
**Time Event**	1.19, 0.49	1.00, 0.63
**Informed Consent**	1.23, 0.55	3.00, 0
**Fidelity of NL to Axiom (% Yes)**	99	63
**People**	100	100
**Time Event**	100	90
**Informed Consent**	97	0
**Fidelity of Axiom to Domain** (**μ****,σ**)	1.13, 0.58	1.3, 0.73
**People**	1.00, 0	1.0, 0
**Time Event**	1.06, 0.29	1.2, 0.63
**Informed Consent**	1.34, 0.69	3.0

## Footnotes

1 http://www.w3.org/TR/owl2-overview/

2 https://www.w3.org/TR/2014/REC-rdfl1-concepts-20140225/

3 https://www.w3.org/TeamSubmission/turtle/

4 https://goo.gl/sGkYli

5 previous called “SEMS” (Semiotic Evaluation Management System)
